# Evaluation of Inhibitory Effect and Mechanism of Euphorbia Factor L_3_ against *Phytophthora capsici*

**DOI:** 10.3390/molecules28072958

**Published:** 2023-03-26

**Authors:** Bi Wang, Guodong Zhang, Jingjing Yang, Linwei Li, Pirui Li, Shu Xu, Xu Feng, Yu Chen

**Affiliations:** Jiangsu Key Laboratory for the Research and Utilization of Plant Resources, Jiangsu Province Engineering Research Center of Eco-Cultivation and High-Value Utilization of Chinese Medicinal Materials, Institute of Botany, Jiangsu Province and Chinese Academy of Sciences (Nanjing Botanical Garden Mem. Sun Yat-Sen), Nanjing 210014, China

**Keywords:** baseline sensitivity, botanical fungicide, Euphorbia factor L_3_, action mechanism, *Phytophthora capsici*

## Abstract

*Phytophthora capsici* is a highly destructive phytopathogenic oomycete with a broad host range and is responsible for tremendous losses. Euphorbia factor L_3_ (EFL_3_) is a natural plant-derived compound that has been widely studied in medicine and cosmetic applications. In this study, the sensitivity of 105 *P. capsici* isolates to EFL_3_ was determined, and the biological activity and physiological effects of EFL_3_ against *P. capsici* were investigated. The median effective concentration (EC_50_) values for EFL_3_ inhibition mycelial growth and spore germination ranged from 0.66 to 8.94 μg/mL (mean, 2.96 ± 0.91 μg/mL) and 1.63 to 13.16 μg/mL (mean, 5.30 ± 1.64 μg/mL), respectively. EFL_3_ treatment resulted in cell wall and cell membrane damage of *P. capsici*, which was revealed by morphological and ultrastructural observations, propidium iodide (PI) and calcofluor white (CFW) staining, and measurements of relative conductivity as well as malondialdehyde (MDA) and glycerol contents. In addition, the contents of phospholipid and cellulose, which are the major components of cell membrane and cell wall, were significantly reduced following EFL_3_ treatment. Furthermore, EFL_3_ provided protective as well as curative efficacies against *P. capsici* on detached tomato leaves and pepper seedlings in vivo. These data show that EFL_3_ exhibits strong inhibitory activity against *P. capsici*, thereby suggesting that it could be an effective alternative for controlling *P. capsici*-induced diseases.

## 1. Introduction

*Phytophthora capsici* Leonian is a notoriously harmful pathogen that can infect more than 60 species of vegetable crops (e.g., pepper, tomato, eggplant, and some melon crops) in the Solanaceous, Cucurbitaceous, and Fabaceous families [1,2,3]. Typical symptoms caused by *P. capsici* on vegetables include stem and fruit rot, wilting, stunting, damping off, and plant death, along with stem and leaf blight [4,5]. It is estimated that *P. capsici*-induced diseases are responsible for at least $200 million in losses each year [6]. As a soilborne pathogenic oomycete, *P. capsici* can survive in the soil for a long time, which leads to difficulties in prevention and control [1,2].

At present, *P. capsici* control mainly depends on synthetic chemical fungicides, such as mefenoxam and metalaxyl. However, long-term use of the existing fungicides has led to the emergence of fungicide-resistant *P. capsici*, which has resulted in control failures [7,8]. In addition, there is considerable concern regarding the adverse effects that some chemical fungicides have on the environment and food safety [9]. Therefore, there is an urgent need to develop natural, safe, and effective antifungal substances for *P. capsici* control. In this sense, plant natural products with antifungal properties are an ideal choice because of their low toxicity and environmental sustainability. Many plant natural products have been reported to possess promising antifungal properties and are recommended for use in controlling plant pathogens. For example, poacic acid inhibits the growth of the fungi *Sclerotinia sclerotiorum* and *Alternaria solani* as well as the oomycete *Phytophthora sojae* [10]. Further, hinokitiol efficiently suppresses the growth of *Alternaria alternata* and *Botrytis cinerea* in postharvest disease measures [11]. More recently, we reported that 10-deacetylbacatin III exhibits effective inhibition against various oomycetes [12].

**Figure 1:**
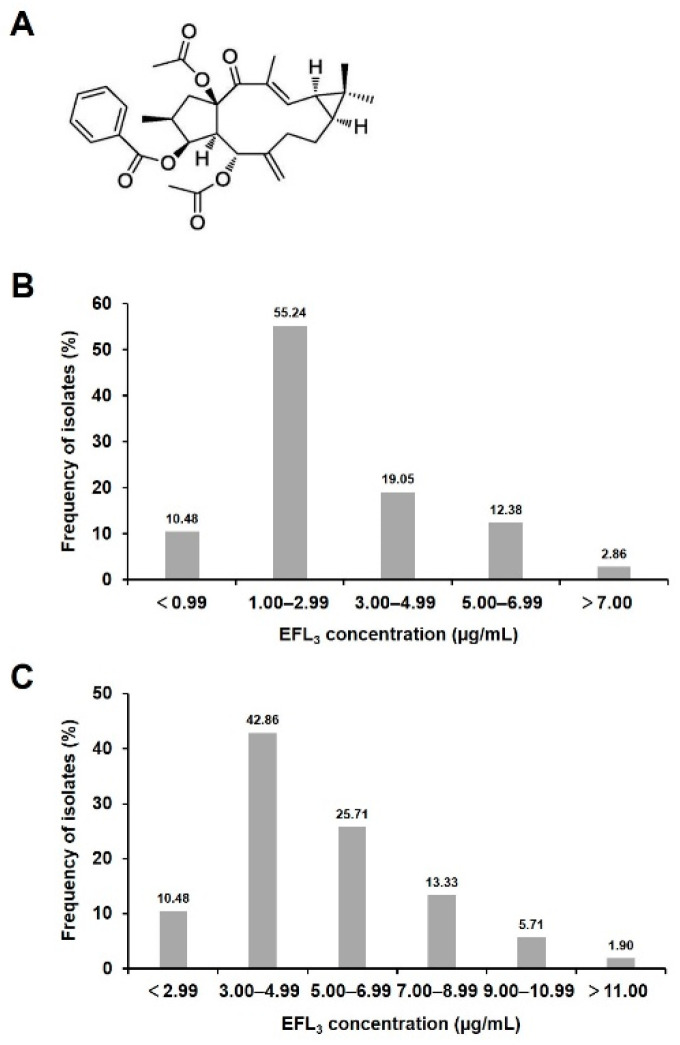
Sensitivity distribution of 105 isolates of *Phytophthora capsici* to Euphorbia factor L_3_ (EFL_3_). (**A**) Chemical structure of EFL_3_. (**B**) Sensitivity distribution of EFL_3_ median effective concentration (EC_50_) values against *P. capsici* from mycelial growth assay. (**C**) Sensitivity distribution of EFL_3_ EC_50_ values against *P. capsici* from spore germination assay.

Euphorbia factor L_3_ (EFL_3_, Figure 1A) is a lathyrane diterpenoid with a 5/11/3-membered ring system [13,14]. It is a natural product isolated mainly from numerous plants from the Euphorbiaceae family, genus *Euphorbia*, which are mostly distributed in Africa, Southeast Asia, and Central to South America [15]. EFL_3_ is one of the main diterpenoids in the seeds of *E. lathyris*, which accounts for 40% of the total diterpenoids in the seeds [16]. The high content of EFL_3_ facilitates its large-scale production and application. Recent studies have revealed that EFL_3_ has a variety of biological activities, such as anti-inflammatory, antitumor, reversing multidrug resistance of tumor cells, and skin whitening [14,17,18,19]. However, there have been no studies into the potential of EFL_3_ as a control agent against phytopathogens and its potential mechanism(s) of action.

Therefore, the aims of this study were to (i) investigate the baseline sensitivity of EFL_3_ against *P. capsici* collected from Jiangsu, Shandong, and Anhui Provinces in China; (ii) explore the mechanism(s) of action of EFL_3_ by biochemical and physiological analyses; and (iii) determine the control effect of EFL_3_ against *P. capsici* on detached tomato leaves and by pot experiments. The results of this study may provide an efficient alternative approach for controlling *P. capsici*-induced diseases.

## 2. Results

### 2.1. Baseline Sensitivity of P. capsici to EFL_3_ by Mycelial Growth and Spore Germination

The sensitivity of *P. capsici* to EFL_3_ was determined using 105 isolates collected from different areas of Jiangsu, Anhui, and Shandong Provinces in 2020 and 2021 (Table S1). The individual EC_50_ values of EFL_3_ for inhibiting mycelial growth ranged from 0.66 to 8.94 μg/mL, with an average value of 2.96 ± 0.91 μg/mL. EFL_3_-resistant populations to EFL_3_ were not identified among these strains, and the frequency distribution of these EC_50_ values was unimodal over a sensitive range (Figure 1B). The range-of-variation factor (maximum/minimum EC_50_ value) was 13.55.

To further determine the effect of EFL_3_ on the secondary infection of *P. capsici*, the sensitivity of EFL_3_ against spore germination of *P. capsici* isolates was tested. The results showed that the individual EC_50_ values for EFL_3_-mediated inhibition of spore germination ranged from 1.63 to 13.16 μg/mL, with an average value of 5.30 ± 1.64 μg/mL, which was approximately 1.8 times the corresponding value for mycelial growth. The frequency distribution of EC_50_ values also formed a unimodal curve, and the range-of-variation factor was 8.07 (Figure 1C). These results indicate that EFL_3_ has a strong inhibitory effect on mycelial growth and spore germination of *P. capsici*. In addition, among the 105 isolates of *P. capsici*, a metalaxyl-resistant strain (TA54, isolated from a host pepper plant in Tai’an, Shandong Province, China) was selected for studies on inhibitory activity and EFL_3_ mechanism.

### 2.2. EFL_3_ Altered P. capsici Morphology and Ultrastructure

**Figure 2:**
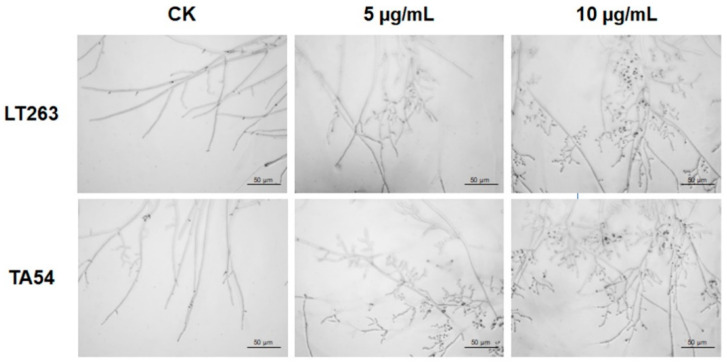
Optical microscopy observations of the mycelial morphology of *P. capsici*. Mycelia of *P. capsici* isolates LT263 and TA54 treated with EFL_3_ at 0, 5, or 10 μg/mL. The scale bar represents 50 μm.

The changes in mycelial morphology of *P. capsici* were clearly shown by optical microscopy (OM) observations. The mycelia of LT263 (the standard strain) and TA54 (a metalaxyl-resistant strain) without exposure to EFL_3_ were uniform, regular, and robust, with a smooth surface and constant diameter. However, after treatment with 5 μg/mL of EFL_3_, the mycelia became swollen and contorted and produced more abnormal branches. Moreover, after treatment with high concentration (10 μg/mL) of EFL_3_, the mycelia became more obviously shrunken and distorted, and the offshoots at the top became shorter (Figure 2).

**Figure 3:**
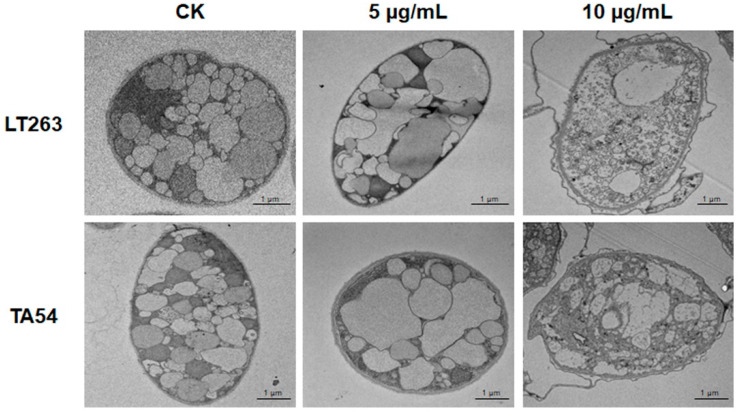
Transmission electron microscopy observations of the mycelial ultrastructure of *P. capsici*. Mycelia of *P. capsici* isolates LT263 and TA54 treated with EFL_3_ at 0, 5, or 10 μg/mL. The scale bar represents 1 μm.

Transmission electron microscopy (TEM) observations were further performed to evaluate the effect of EFL_3_ on mycelial ultrastructure of *P. capsici*. The mycelia of LT263 and TA54, grown in the absence of EFL_3_, contained a uniform cytoplasmic matrix and intact cellular organelles. The plasma membrane and cell wall were smooth and intact. By contrast, the normal ultrastructure of *P. capsici* was destroyed in the EFL_3_ treatment group. After exposure to 5 μg/mL of EFL_3_, the thickness of the plasma membrane and cell wall decreased, the vasculature increased, and the cellular organelles began to degrade. High concentrations of EFL_3_ (10 μg/mL) caused greater damage to the mycelial ultrastructure. The plasma membrane and cell wall ruptured; cell vacuolation was greater; and cellular organelles, such as nucleus and mitochondria, completely disappeared (Figure 3). These data suggest that EFL_3_ causes severe disruption of morphology and ultrastructure of *P. capsici*, leading to cell death.

### 2.3. EFL_3_ Affected the Cell Membrane Permeability of P. capsici

**Figure 4:**
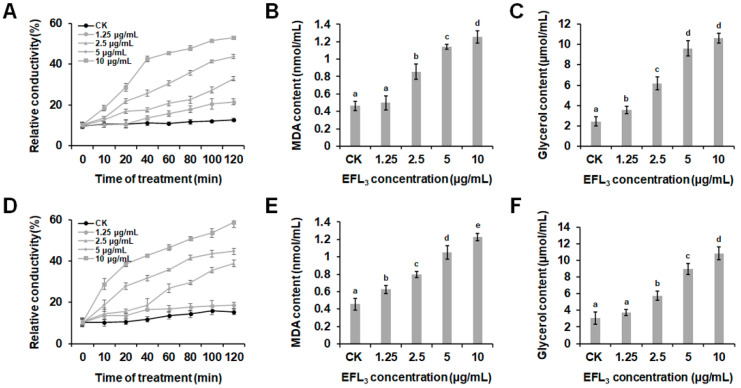
Effect of EFL_3_ on the cell membrane permeability of *P. capsici*. (**A**,**D**) Effect of EFL_3_ on the relative electrical conductivity of *P. capsici* isolates LT263 and TA54. (**B**,**E**) Effect of EFL_3_ on the malondialdehyde (MDA) content of *P. capsici* isolates LT263 and TA54. (**C**,**F**) Effect of EFL_3_ on the glycerol content of *P. capsici* isolates LT263 and TA54. Data are shown as the mean ± standard error (SE), and small letters indicate significant differences across treatments (*p* < 0.05).

The effect of EFL_3_ on the cell membrane permeability of *P. capsici* was investigated by measuring the extracellular conductivity as well as malondialdehyde (MDA) and glycerol contents of *P. capsici*. As shown in Figure 4A,D, after treatment with EFL_3_, the relative conductivities of LT263 and TA54 were always higher than the untreated control. The relative conductivity increased with increasing EFL_3_ concentrations and times. After incubation for 120 min, the relative conductivities of LT263 and TA54 in 10 μg/mL of EFL_3_ treatment group were 4.18 and 3.81 times higher, respectively, than that of the untreated control (Figure 4A,D).

As illustrated in Figure 4B,E, treatment with EFL_3_ resulted in a remarkable increase in the MDA content of both LT263 and TA54 compared to the control. This response was also dose dependent. Similar results were observed for glycerol content. The addition of EFL_3_ induced a significant increase in the glycerol content of LT263 and TA54, while the levels in the controls remained stable during the whole period. In addition, the glycerol content also increased as the EFL_3_ concentration increased (Figure 4C,F). These results suggest that EFL_3_ may target cell membrane permeability of *P. capsici*.

### 2.4. EFL_3_ Destroyed the Cell Membrane and Cell Wall Integrity of P. capsici

**Figure 5:**
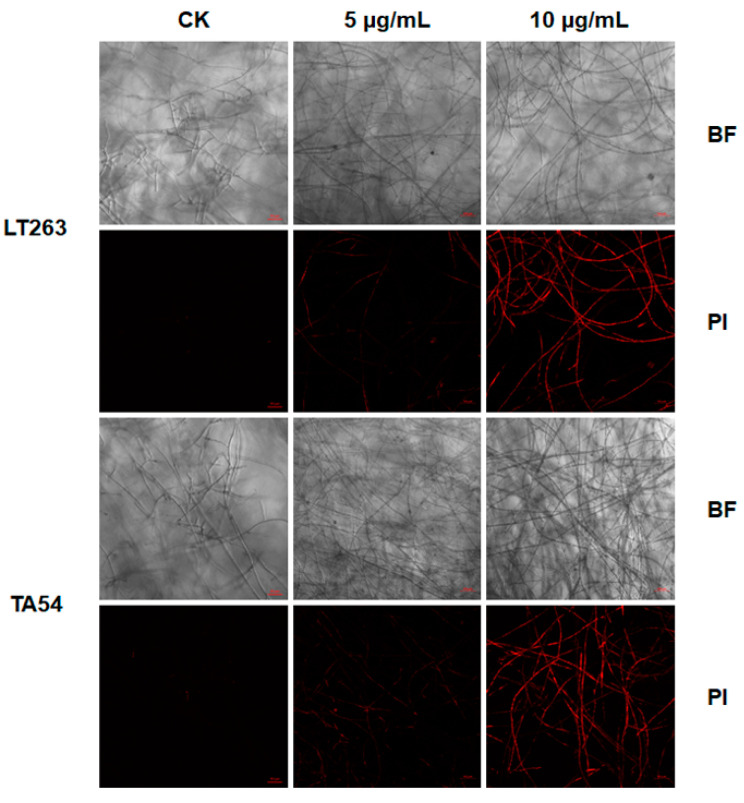
Effect of EFL_3_ on the cell membrane integrity of *P. capsici*. The mycelia of *P. capsici* isolates LT263 and TA54 were stained with propidium iodide (PI) and observed under a fluorescence microscope. The scale bar represents 50 μm.

To further explore the potential mechanisms by which EFL_3_ exhibits antioomycete activity, propidium iodide (PI) and calcofluor white (CFW) straining were used to determine cell membrane and cell wall integrity. PI is a fluorescent dye that is often used to indicate cell membrane damage [20]. As shown in Figure 5, the mycelia of LT263 and TA54 in the untreated control showed no detectable fluorescence, presumably because PI was unable to penetrate mycelia with an intact membrane. However, the mycelia showed a bright red fluorescence after exposure to EFL_3_. 

**Figure 6:**
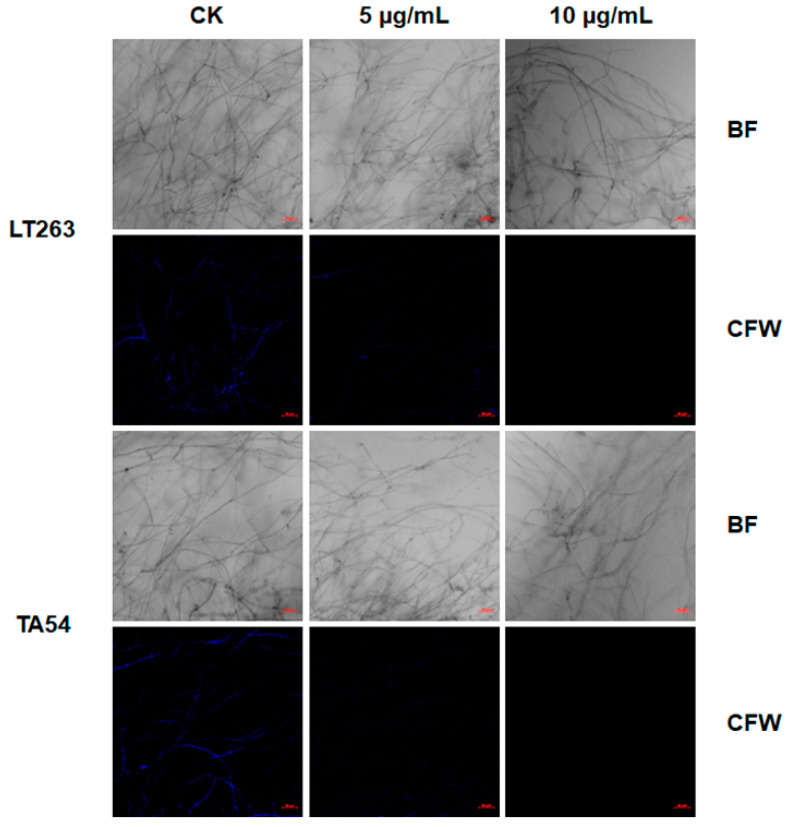
Effect of EFL_3_ on the cell wall integrity of *P. capsici*. The mycelia of *P. capsici* isolates LT263 and TA54 were stained with calcofluor white (CFW) and observed under a fluorescence microscope. The scale bar represents 50 μm.

CFW is a fluorescent brightener that has strong affinity to cellulose in the cell wall of various plants and oomycetes [21]. Here, the effect of EFL_3_ on cell wall integrity was analyzed by CFW staining. As shown in Figure 6, EFL_3_ treatment significantly affected the cellulose distribution of both LT263 and TA54. The blue fluorescence of mycelia in the EFL_3_ treatment group was weaker than the untreated control. All these results indicate that EFL_3_ impairs cell membrane and cell wall integrity of *P. capsici*.

### 2.5. EFL_3_ Affected the Phospholipid and Cellulose Contents of P. capsici

**Figure 7:**
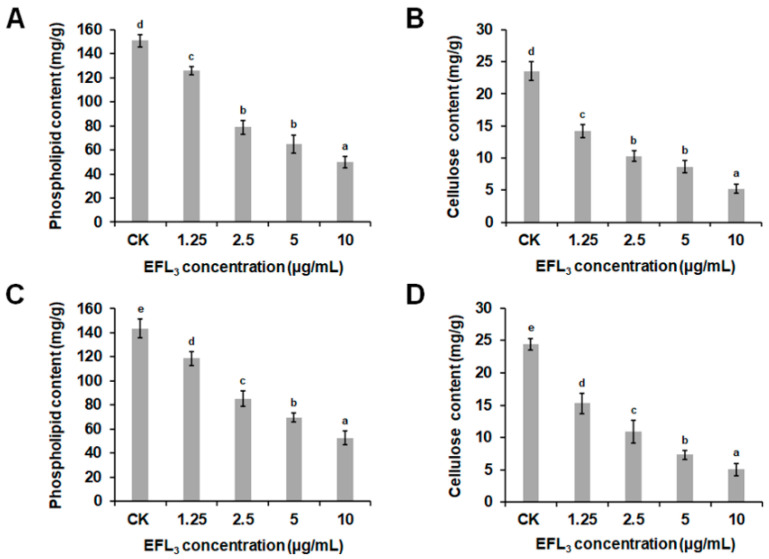
Effect of EFL_3_ on phospholipid and cellulose contents of *P. capsici*. (**A**,**C**) Effect of EFL_3_ on phospholipid content of *P. capsici* isolates LT263 and TA54. (**B**,**D**) Effect of EFL_3_ on cellulose content of *P. capsici* isolates LT263 and TA54. Data are shown as the mean ± standard error (SE), and small letters indicate significant differences across treatments (*p* < 0.05).

Phospholipids are the main components of the oomycete cell membrane, which is very important for maintaining cell membrane integrity and function [22,23]. In this study, the phospholipid content in the mycelia of LT263 and TA54 were detected. As shown in Figure 7A,C, the phospholipid content of *P. capsici* was significantly lower than that of the control group after exposure to different concentrations of EFL_3_. Compared to the untreated control, treatment with EFL_3_ at 1.25, 2.5, 5, and 10 μg/mL resulted in a decrease in the phospholipid content of LT263 and TA54 by 16.52, 47.60, 56.95, and 67.06% and 17.43, 40.60, 51.46, and 63.42%, respectively.

Cellulose acts as the structural backbone of the oomycete cell wall [24]. To further establish the effect of EFL_3_ on the cell wall, the cellulose content of *P. capsici* was also determined. The cellulose content of LT263 and TA54 incubated with 1.25, 2.5, 5, and 10 μg/mL of EFL_3_ were 14.21, 10.28, 8.67, and 5.23 mg/g and 15.28, 10.90, 7.31, and 5.04 mg/g, respectively, which were significantly lower than that of the untreated control (23.54 and 24.42 mg/g) (Figure 7B,D). Hence, EFL_3_ may reduce the phospholipid and cellulose contents of *P. capsici* to further affect the integrity of the cell membrane and cell wall.

### 2.6. Curative and Protective Activities of EFL_3_ against P. capsici

**Figure 8:**
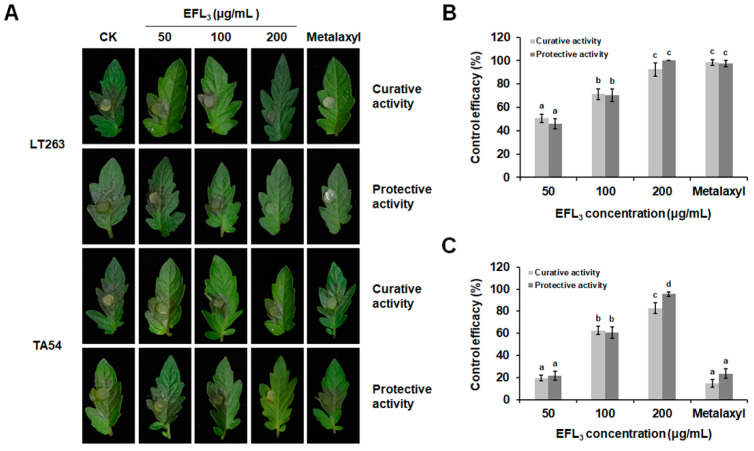
Control activity of EFL_3_ against *P. capsici* on detached tomato leaves. (**A**) Disease symptoms on tomato leaves were photographed after inoculation with a fresh mycelial plug for 4 days. (**B**) Protective and curative activities of EFL_3_ against *P. capsici* isolate LT263. (**C**) Protective and curative activities of EFL_3_ against *P. capsici* isolate TA54. Data are shown as the mean ± standard error (SE), and small letters indicate significant differences across treatments (*p* < 0.05).

To understand the control efficacy of EFL_3_ against *P. capsici*, the protective and curative activities of EFL_3_ on detached leaves of tomato were determined. Tomato leaf inoculation assays showed that EFL_3_ exhibited strong control efficacy on the diseases caused by *P. capsici*. As shown in Figure 8A, EFL_3_ reduced the symptoms caused by LT263 and TA54 in a dose-dependent manner. The curative and protective efficacies of EFL_3_ were 50.64 and 45.76% for LT263 and 19.54 and 21.65% for TA54, respectively, after treatment with 50 μg/mL of EFL_3_. When the concentration of EFL_3_ reached 200 μg/mL, its curative and protective efficacies were 92.47 and 100% for LT263 and 82.68 and 95.49% for TA54, respectively. Moreover, the curative and protective efficacies of 200 μg/mL metalaxyl against LT263 were 98.86 and 96.52%. In contrast, the curative and protective efficacies of 200 μg/mL metalaxyl against TA54 was much lower (only 14.70 and 23.51%, respectively; Figure 8B,C). 

**Figure 9:**
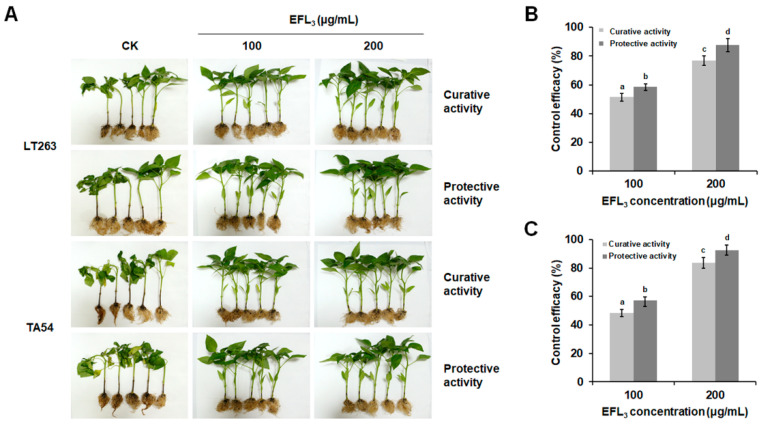
Control activity of EFL_3_ against *P. capsici* on pepper seedlings. (**A**) Disease symptoms on pepper seedlings were photographed after being injected with zoospore suspensions for 10 days. (**B**) Protective and curative activities of EFL_3_ against *P. capsici* isolate LT263. (**C**) Protective and curative activities of EFL_3_ against *P. capsici* isolate TA54. Data are shown as the mean ± standard error (SE), and small letters indicate significant differences across treatments (*p* < 0.05).

Given that *P. capsici* is a soilborne pathogen, to further clarify the control efficacy of EFL_3_ against *P. capsici*, additional pot experiments were conducted on pepper seedlings. Similarly, as shown in Figure 9A, the disease symptoms on pepper seedlings caused by *P. capsici* were significantly inhibited by EFL_3_ treatment. After exposure to 100 μg/mL of EFL_3_, the curative and protective efficacies were 51.36 and 58.52% for LT263 and 48.52 and 57.36% for TA54, respectively. When the concentration of EFL_3_ reached 200 μg/mL, the curative and protective efficacies of EFL_3_ were 76.73 and 87.64% for LT263 and 83.54 and 92.42% for TA54, which was consistent with the results of the detached leaf assay. Notably, the protective activity of EFL_3_ against *P. capsici* was always superior than the curative activity within the same treatment concentration (Figure 9B,C). Overall, these results suggest that EFL_3_ may be a promising fungicide against diseases caused by *P. capsici*.

## 3. Discussion

*P. capsici* is a highly destructive phytopathogenic oomycete with a broad host range [1,2,3]. At present, fungicide application is still the main method for controlling diseases caused by *P. capsici*. The long-term use of conventional fungicides, however, has increased the development of fungicide-resistant pathogens [7,8]. For these reasons, chemical substitutes with different modes of action and high inhibitory activity are urgently needed. EFL_3_ is a lathyrane diterpenoid, which is a natural product mainly isolated from Euphorbiaceae plants [13,14]. In recent years, the medical and cosmetic values of EFL_3_ have been extensively reported, but its antifungal activities against phytopathogens have not been reported yet [14,17,18,19]. In this study, we evaluated EFL_3_-mediated control of diseases caused by *P. capsici*.

Baseline sensitivity is very important for monitoring the occurrence of fungicide resistance. Hence, it is necessary to determine the baseline sensitivity of the pathogens to fungicide before its legal use [25]. In this study, baseline sensitivity to EFL_3_ was first determined by measuring the sensitivity of 105 isolates of *P. capsici* collected from Jiangsu, Anhui, and Shandong Provinces in China. The baseline sensitivity curves of EFL_3_ were unimodal with average EC_50_ values of 2.96 ± 0.91 and 5.30 ± 1.64 μg/mL for inhibition of mycelial growth and spore germination, respectively, while the range-of-variation factors were 13.55 and 8.07, respectively. The low EC_50_ values and narrow range-of-variation factors suggest that no EFL_3_-resistant isolates were found in our study. Furthermore, the average EC_50_ values for EFL_3_ were lower than the previously published EC_50_ values of other plant-derived natural products, such as cinnamaldehyde and esculetin, against *P. capsici* [26,27]. These data show that EFL_3_ has a strong inhibitory effect on mycelial growth and spore germination of *P. capsici*.

Given the excellent inhibitory effect of EFL_3_ against *P. capsici*, we evaluated the physiological and biochemical effects of EFL_3_ against *P. capsici* to explore antifungal mechanisms. The antifungal activity of plant-derived terpenoids has been partly attributed to their destructive effects on pathogen cell wall and cell membrane. For example, thymol was reported to lead to the loss of cell morphology, irregular shrinkage, and even serious fracture of hyphae of *Botrytis cinerea* [28]. In this study, we found that EFL_3_ damaged the morphological and ultrastructural characteristics of *P. capsici*. In addition, EFL_3_ treatment caused increased electrical conductivity and upregulated MDA and glycerol contents. Furthermore, using PI and CFW straining, we found that EFL_3_ severely disrupted the cell membrane and cell wall integrity of *P. capsici*, which eventually led to cell death. Taken together, these results suggest that the antifungal mechanism of EFL_3_ may be attributed to its destructive potential in the cell membrane and cell wall.

Phospholipids are major components of the oomycete cell membrane, which is very important for maintaining cell membrane integrity and function [22,23]. Cellulose acts as the structural backbone of the oomycete cell wall [24]. Many fungicides have been reported to destroy the cell membrane or cell wall by inhibiting their main structural components. For example, clotrimazole and itraconazole achieved their fungicidal effects against citrus green mold by causing a decrease in ergosterol content, which subsequently affected cell membrane integrity and function [29,30]. Li et al. (2021) showed that carvacrol significantly decreased the total lipid and ergosterol contents to disrupt the cell membrane in the hyphae of *Botryosphaeria dothidea* [31]. In our study, phospholipid and cellulose contents decreased significantly following EFL_3_ treatment, which would explain the observed destruction of the cell membrane and cell well. However, the mechanism by which EFL_3_ causes the decrease in phospholipid and cellulose contents in *P. capsici* remains unclear. Thus, further investigations are required to elucidate this phenomenon.

Fungicides with both protective and curative effects can effectively control disease occurrence and prevalence [32]. In this study, we investigated the protective and curative activities of EFL_3_ against *P. capsici* on detached tomato leaves and by pot experiments. The results indicated that EFL_3_ displayed both protective and curative activities against *P. capsici* on detached tomato leaves and pepper seedlings. Moreover, the protective activity of EFL_3_ was better than the curative activity at the same concentration on pepper seedlings. This suggests that EFL_3_ should be used prophylactically or in the early stage of disease for better control. In addition, the control effect of EFL_3_ against *P. capsici* seemed to be more effective than that of other plant-derived natural products, such as liquiritin and cinnamaldehyde [27,32]. Our data suggest that EFL_3_ has the potential to be an excellent alternative fungicide to control diseases caused by *P. capsici*. To further confirm the control efficacy of EFL_3_ against *P. capsici*, field experiments will be carried out in the near future. The antifungal mechanism of EFL_3_ may be attributed to its destructive potential in the cell membrane and cell wall. Because the composition of the cell membrane and cell wall is similar in plants and oomycetes [33,34], EFL_3_ may have some effects on nontarget organisms (plants). In this study, we use the inoculation assay on both tomato leaves and pepper seedlings by treating them with different concentrations of EFL_3_. However, as shown in Figure 8 and Figure 9, there was no visible effect of EFL_3_ on tomato leaves and pepper seedlings. The effect of EFL_3_ on the fruit quality of tomato and pepper will be determined in further studies.

## 4. Materials and Methods

### 4.1. General Experimental Procedures

Column chromatography was performed on silica gel (200–300 mesh; Qingdao Haiyang Chemicals, Qingdao, China) and Sephadex LH-20 (Amersham Pharmacia Biotech AB, Staffanstorp, Sweden) columns. Preparative HPLC (LC-20AR, Shimadzu, Japan) was conducted using a YMC-Pack ODS-AQ column (5 μm, 250 × 20 mm, Shimadzu). LC–HRMS spectra were obtained on an Agilent 6546 Q-TOF mass spectrometer coupled to an Agilent 1290 Infinity UPLC instrument (Agilent Technologies GmbH, Waldbronn, Germany). NMR spectra were recorded on Bruker AVANCE-300 NMR spectrometer, and CDCl_3_ was used as solvent and TMS as an internal standard (*δ* in ppm, *J* in Hz).

### 4.2. Chemicals

EFL_3_ was isolated from *Euphorbia lathyris* as previously reported [35]. Briefly, seeds of *E. lathyris* (2 kg) were refluxed with 95% (*v*/*v*) aqueous ethanol. After filtration, the solution was concentrated under reduced pressure to make a residue (230.4 g), which was suspended in distilled water and successively partitioned with petroleum ether, dichloromethane (DCM), and ethyl acetate. The DCM extract was separated and repeatedly purified by silica gel and HPLC column chromatography to afford EFL_3_ (400 mg). EFL_3_: white powder; HR-(+)-EIMS *m*/*z*: 545.2497 [M+Na]^+^ (calculated for C_31_H_38_O_7_Na, 545.2510); ^1^H-NMR (CDCl_3_, 300 MHz): *δ* 0.94 (3H, d, *J* = 6.7 Hz), 1.16 (1H, m), 1.18 (3H, s), 1.19 (3H, s), 1.28 (3H, s), 1.41 (1H, dd, *J* = 8.3, 11.2 Hz), 1.66 (1H, dd, *J* = 11, 14 Hz), 1.68 (1H, m), 1.78 (3H, s), 1.91 (1H, m), 2.01 (1H, m), 2.15 (1H, m), 2.24 (3H, s), 2.36 (1H, m), 2.92 (1H, dd, *J* = 3.4, 8.7 Hz), 3.52 (1H, dd, *J* = 8, 14 Hz), 4.91 (1H, s), 5.08 (1H, s), 5.82 (1H, t, *J* = 3.2 Hz), 6.29 (1H, d, *J* = 9.9 Hz), 6.54 (1H, d, *J* = 11.1 Hz), 7.59 (1H, t, *J* = 7.5 Hz), 7.61 (2H, t, *J* = 7.8 Hz), 8.09 (2H, d, *J* = 7.2 Hz); ^13^C-NMR (CDCl_3_, 75 MHz): *δ* 12.6, 14.3, 17.0, 21.1, 21.8, 22.1, 25.4, 28.7, 29.1, 35.1, 35.5, 38.1, 48.7, 52.4, 65.7, 81.0, 92.7, 115.5, 128.4, 129.8, 130.3, 133.2, 134.4, 144.8, 146.5, 166.3, 169.8, 170.2, 196.9 (Figures S1–S4).

Metalaxyl (98%) was purchased from Aladdin (Shanghai, China). EFL_3_ and metalaxyl were dissolved in dimethyl sulfoxide (DMSO) to obtain a stock solution of 10 mg/mL. Other chemicals and reagents of analytical grade were purchased from Solarbio (Beijing, China).

### 4.3. P. capsici Isolates and Culture Conditions

Isolates (n = 105) of *P. capsici* from pepper, tomato, eggplant, and cucumber were collected from different areas of Jiangsu, Anhui, and Shandong Provinces in 2020 and 2021 (Table S1). The sampling fields had never been exposed to metalaxyl before, and they were at least 20 km apart from each other. In each region, samples (leaf, stem, fruit, or root) with typical symptoms of *P. capsici* infection were collected and cut into small pieces (3 × 3 mm). All pieces were surface sterilized using 2% NaClO (*v*/*v*), washed with sterilized distilled water, and then placed on V8 juice agar plates and incubated for 4 days at 25 °C. *P. capsici* isolates were identified by colony morphology, sporangium sharp, and sequence analysis [36]. Among the 105 *P. capsici* isolates, a metalaxyl-resistant strain (TA54, isolated from a host pepper plant in Tai’an, Shandong Province, China) was selected for studies on inhibitory activity and EFL_3_ mechanism.

### 4.4. Determination of Baseline Sensitivity of P. capsici to EFL_3_

The baseline sensitivity of *P. capsici* to EFL_3_ was determined using the mycelial growth inhibition method [26]. The test *P. capsici* isolates (n = 105) were cultivated on V8 juice agar plates at 25 °C for 4 days to generate new mycelia. Next, fresh mycelial plugs were cut and incubated on V8 juice agar plates supplemented with 0, 1.25, 2.5, 5, 10, or 20 μg/mL of EFL_3_. After incubation for 4 days, the colony diameters were measured and growth inhibition rates were calculated. The median effective concentration (EC_50_) values were calculated based on linear regression of the colony diameter on log-transformed EFL_3_ concentration [37].

The baseline sensitivity of *P. capsici* to EFL_3_ by spore germination was determined as previously reported [26]. The test *P. capsici* isolates (n = 105) were again cultivated on V8 juice agar plates at 25 °C for 4 days to generate new mycelia. Then, 10 fresh mycelial plugs were cut and incubated on a plate that contained 10 mL of V8 juice medium. After incubation for 2 days at 25 °C, the mycelia were washed and resuspended in sterilized tap water in the dark at 25°C for 1 day for sporangial formation. Then, the cultures were transferred to 4 °C for 1 h followed by incubation at 25 °C to release zoospores from sporangia. Zoospore suspensions (1 × 10^5^ spores/mL) were exposed to different concentrations of EFL_3_ and then incubated at 25 °C in the dark for 12 h. Germinated spores were counted at three random sites on each plate under a microscope. The spore germination inhibition rate was calculated. EC_50_ values were calculated based on linear regression on log-transformed EFL_3_ concentration [38]. The bioassay data were obtained from three independent biological replicates.

### 4.5. OM and TEM Observations

The effects of EFL_3_ on the mycelial morphology and ultrastructure of *P. capsici* were observed using OM and TEM observations [39]. Fresh mycelial plugs of LT263 (standard strain of *P. capsici*) and TA54 were cut and incubated on V8 juice agar plates supplemented with 0, 5, or 10 μg/mL of EFL_3_. After incubation for 2 days, the mycelia were taken from the periphery of the colonies and placed on glass slides. The *P. capsici* mycelial morphology was observed, and images were obtained using OM. For TEM, the samples were fixed in 4% (*v*/*v*) glutaraldehyde at 4°C for 6 h and dissolved in 1% (*v*/*v*) osmium tetroxide at room temperature for 2 h. Samples were then subjected to a graded ethanol series (30, 50, 70, 90, and 100% *v*/*v*) for 15 min at each stage for dehydration and embedded in Epon resin. After that, the samples were cut into ultrathin sections followed by staining with 2% (*v*/*v*) uranyl acetate and lead citrate. The mycelial ultrastructure of *P. capsici* was observed and photographed using a transmission electron microscope (Talos-F200C, Thermo Fisher Scientific, Waltham, MA, USA). Experiments were performed with three biological replicates, each with three technical replicates.

### 4.6. Effect of EFL_3_ on Cell Membrane Permeability of P. capsici

The relative conductivity of *P. capsici* mycelia was measured following the conditions reported before [40]. First, 10 fresh mycelial plugs of LT263 and TA54 were cut and incubated into a flask that contained 100 mL of V8 juice medium. After incubation for 2 days at 25 °C with shaking at 175 rpm, EFL_3_ at final concentrations of 0, 1.25, 2.5, 5, or 10 μg/mL was added to the flasks. After incubation for another day at 25 °C with shaking at 175 rpm, the mycelia were washed and resuspended in sterilized distilled water. Afterwards, the conductivity of the suspension was measured with a conductivity meter (DDS-11A, Shanghai Leici Instrument Inc., Shanghai, China) at 0, 10, 20, 40, 80, 100, and 120 min. After 120 min, all samples were boiled, and the final conductivity measurement was made. Relative conductivity (%) = (conductivity/final conductivity) × 100.

The effects of EFL_3_ on MDA and glycerol contents in *P. capsici* mycelia were evaluated as described by Wang et al. [41]. Mycelia were cultured as described above. After being collected and washed with sterilized distilled water, mycelia samples were ground with liquid nitrogen and dissolved in PBS (pH 7.2). The supernatant was collected by centrifugation at 5000× *g* for 20 min. The MDA and glycerol contents of *P. capsici* mycelia were measured using MDA (Nanjing Jiancheng Bioengineering Institute, Nanjing, China) and glycerol (Beijing Applygen Technologies Inc., Beijing, China) detection kits. Three biological replicates were obtained.

### 4.7. Effect of EFL_3_ on P. capsici Cell Wall and Cell Membrane Integrity

The effect of EFL_3_ on cell wall integrity was measured as described before [26,42]. In brief, fresh mycelial plugs of LT263 and TA54 were cut and incubated into a plate containing 10 mL of V8 juice medium. After incubation for 1 day at 25 °C, EFL_3_ at final concentrations of 0, 5, or 10 μg/mL was added to the plates. After incubation for another day at 25°C, the mycelia were washed with PBS (pH 7.2) and stained with 10 μL of 1% (*v*/*v*) CFW and 10 μL of 10% (*v*/*v*) KOH for 1 min. The mycelia were then washed with PBS again and observed under a fluorescence microscope (LSM880, Carl Zeiss, Jena, Germany). The effect of EFL_3_ on cell membrane integrity was detected using PI staining. Mycelia were cultured as described above. After incubation with different concentrations of EFL_3_ for 1 day, the mycelia were washed with PBS and then stained with 50 μg/mL PI for 20 min in the dark. After that, the mycelia were rinsed with PBS again and observed under a fluorescence microscope. The experiment was repeated three times.

### 4.8. Effect of EFL_3_ on P. capsici Phospholipid and Cellulose Contents

The effect of EFL_3_ on phospholipid content was measured following the methods reported before [22]. Mycelia were cultured as described above in Section 2.6. After being collected and washed with sterilized distilled water, mycelia samples were ground with liquid nitrogen. Subsequently, 10 mg mycelia per sample were collected and resuspended in the assay buffer. The phospholipid content of *P. capsici* mycelia were measured using a phospholipid assay kit (Sigma-Aldrich, St. Louis, MO, USA). The effect of EFL_3_ on cellulose content was also determined. Mycelia were cultured and collected as described above. After being ground with liquid nitrogen, 0.3 g of mycelia per sample were collected and resuspended in the extraction buffer. The cellulose content of *P. capsici* mycelia were measured by a cellulose assay kit (Solarbio, Beijing, China). The experiment was repeated three times.

### 4.9. Control Efficacy of EFL_3_ against P. capsici

The control efficacy of EFL_3_ against *P. capsici* was first tested on tomato leaves as reported before [41]. Tomato leaves (*Solanum lycopersicum* L. cv. ‘Hongbaoshi’) were detached from the tomatoes at the four- to six-leaf stage. Leaves with uniform size, color, and shape were selected after surface sterilization with 2% (*v*/*v*) NaClO. Following surface sterilization, tomato leaves were sprayed with EFL_3_ at final concentrations of 0, 50, 100, and 200 μg/mL or metalaxyl at 200 μg/mL. For protective efficacy determination, the leaves were inoculated with fresh mycelial plugs after being sprayed with EFL_3_ or metalaxyl dilutions for 24 h. For curative efficacy determination, the leaves were first inoculated with fresh mycelial plugs. Then, after 24 h, the surface of each tomato leaf was sprayed with the drugs. Leaves within the same treatment were placed in one sealed plastic container and incubated at 25 °C and a long-day photoperiod. After inoculation for 4 days, the lesion area on the leaves was measured and calculated. Each group consisted of 15 leaves, and the experiment was performed in triplicate. Control efficacy = (lesion area of water treatment − lesion area of drug treatment)/lesion area of water treatment.

Given that *P. capsici* is a soilborne pathogen, to further clarify the control efficacy of EFL_3_ against *P. capsici*, pot experiments were carried out as described by Li et al. [43]. Pepper seedlings (*Capsicum annuum* L. cv. ‘Sujiao 5′) at the four- to six-leaf stage were obtained from vegetable fields in Nanjing, Jiangsu Province, China. Zoospore suspensions (1 × 10^5^ spores/mL) of LT263 and TA54 were prepared as described above in Section 4.4. Pepper seedlings were sprayed with EFL_3_ at final concentrations of 0, 100, and 200 μg/mL. A sterilized needle was used to prick the stem base of each pepper seedling to make a wound site. For protective efficacy determination, the wound site was inoculated with 5 μL of zoospore suspensions after being sprayed with EFL_3_ dilutions for 24 h. For curative efficacy determination, the seedlings were first inoculated with zoospore suspensions. Then, after 24 h, the surface of each pepper seedling was sprayed with the treatments. Seedlings within the same treatment were placed in a growth chamber and incubated at 23–28 °C and a long-day photoperiod. After inoculation for 10 days, the disease severity was recorded using the crown rot system scale (0 to 5, Figure S5). The disease index = Σ(scale × plant numbers). Each group consisted of 20 seedlings, and the experiment was performed in triplicate. Control efficacy = (disease index of water treatment − disease index of drug treatment)/disease index of water treatment.

### 4.10. Statistical Analysis

Data are shown as the mean ± standard error (SE). All statistical analyses were carried out using SPSS software (v14.0 SPSS Inc., Chicago, IL, USA). The significance of the difference between treatments was determined using Fisher’s least significant differences tests at *p* < 0.05 level.

## 5. Conclusions

In conclusion, we have demonstrated that EFL_3_ exhibits strong inhibitory activity against mycelial growth and spore germination of *P. capsici* in vitro and that it is also effective for controlling diseases caused by *P. capsici* in vivo. The antifungal mechanism of EFL_3_ may be attributed to its destructive potential in the cell membrane and cell wall via the inhibition of phospholipid and cellulose biosynthesis. However, further molecular studies are required to establish the molecular mechanisms by which EFL_3_ negatively impacts *P. capsici*.

## Data Availability

Data is contained within the supplementary material (Supplementary Table S1 and Figures S1–S5).

## Supplementary Materials

The following supporting information can be downloaded at: https://www.mdpi.com/article/10.3390/molecules28072958/s1, Table S1: Information on the 105 *Phytophthora capsici* strains used in this study; Figure S1: UPLC chromatograms of EFL_3_; Figure S2: HR-ESI–MS spectrum of EFL_3_; Figure S3: ^1^H-NMR spectrum (300 MHz, CDCl_3_) of EFL_3_; Figure S4: ^13^C-NMR spectrum (75 MHz, CDCl_3_) of EFL_3_; Figure S5: The disease severity of *Phytophthora capsici* on pepper plants.

## Sample Availability

Samples of the compounds are not available from the authors.
