# Supplementary material for: Evaluation of Inhibitory Effect and Mechanism of Euphorbia Factor L3 against Phytophthora capsici
Source: Molecules. 2023 Mar 26;28(7):2958. doi: 10.3390/molecules28072958 (PMC10096396; doi:10.3390/molecules28072958)
Supplement: Supplementary file 1 [file molecules-28-02958-s001.zip › molecules-2276097-supplementary.pdf]

Supplementary data

Evaluation of Inhibitory Effect and Mechanism of Euphorbia Factor L3 against *Phytophthora capsici*

Bi Wang, Guodong Zhang, Jingjing Yang, Linwei Li, Pirui Li, Shu Xu, Xu Feng * and Yu Chen *

Jiangsu Key Laboratory for the Research and Utilization of Plant Resources, Jiangsu Province Engineering Research Center of Eco-Cultivation and High-Value Utilization of Chinese Medicinal Materials, Institute of Botany, Jiangsu Province and Chinese Academy of Sciences (Nanjing Botanical Garden Mem. Sun Yat-Sen), Nanjing 210014, China; wangbi@cnbg.net (B.W.); zgd2134567@163.com (G.Z.); jing5_5@126.com (J.Y.); lilinwei@cnbg.net (L.L.); lipirui011824@yeah.net (P.L.); xxssuu1990@163.com (S.X.)

* Correspondence: fengxu@jib.ac.cn (X.F.); ychen@jib.ac.cn (Y.C.)

Table S1. The information of the 105 *Phytophthora capsici* strains used in this study.

No.	Strains	Origins	Hosts	EC_{50} against mycelial growth (μg/mL)	EC_{50} against spore generation (μg/mL)
1	HA98	Huan'an, Jiangsu Province	Tomato	1.55	3.87
2	HA34	Huan'an, Jiangsu Province	Cucumber	3.07	5.84
3	HA23	Huan'an, Jiangsu Province	Pepper	0.95	3.09
4	HA46	Huan'an, Jiangsu Province	Tomato	4.06	7.14
5	HA147	Huan'an, Jiangsu Province	Tomato	1.34	5.61
6	HA02	Huan'an, Jiangsu Province	Pepper	3.29	6.13
7	HA81	Huan'an, Jiangsu Province	Pepper	2.17	4.69
8	HA105	Huan'an, Jiangsu Province	Cucumber	2.14	4.64
9	HA120	Huan'an, Jiangsu Province	Eggplant	1.98	4.43
10	HA65	Huan'an, Jiangsu Province	Pepper	3.06	5.84
11	HA12	Huan'an, Jiangsu Province	Tomato	1.92	3.61
12	HA101	Huan'an, Jiangsu Province	Cucumber	2.57	4.49
13	XZ90	Xuzhou, Jiangsu Province	Pepper	5.85	8.95
14	XZ97	Xuzhou, Jiangsu Province	Eggplant	1.94	5.63
15	XZ82	Xuzhou, Jiangsu Province	Tomato	2.56	8.48
16	XZ35	Xuzhou, Jiangsu Province	Tomato	0.77	1.63

17	XZ179	Xuzhou, Jiangsu Province	Pepper	2.05	5.78
18	XZ66	Xuzhou, Jiangsu Province	Cucumber	6.29	9.55
19	XZ43	Xuzhou, Jiangsu Province	Cucumber	8.94	13.16
20	XZ161	Xuzhou, Jiangsu Province	Tomato	5.84	8.94
21	XZ70	Xuzhou, Jiangsu Province	Cucumber	1.82	5.19
22	DT84	Dongtai, Jiangsu Province	Tomato	2.94	5.52
23	DT30	Dongtai, Jiangsu Province	Pepper	0.73	1.88
24	DT138	Dongtai, Jiangsu Province	Cucumber	1.95	5.34
25	DT60	Dongtai, Jiangsu Province	Pepper	4.06	5.87
26	DT79	Dongtai, Jiangsu Province	Eggplant	5.26	7.32

**No.**	**Strains**	**Origins**	**Hosts**	**EC_{50} against mycelial growth (μg/mL)**	**EC_{50} against spore generation (μg/mL)**
27	DT135	Dongtai, Jiangsu Province	Pepper	3.63	5.35
28	DT88	Dongtai, Jiangsu Province	Tomato	0.84	2.01
29	DT90	Dongtai, Jiangsu Province	Tomato	2.09	5.50
30	DT123	Dongtai, Jiangsu Province	Pepper	2.89	4.46
31	DT07	Dongtai, Jiangsu Province	Cucumber	2.26	4.06
32	SQ169	Suqian, Jiangsu Province	Tomato	2.80	5.78
33	SQ112	Suqian, Jiangsu Province	Cucumber	0.66	1.89
34	SQ62	Suqian, Jiangsu Province	Pepper	1.84	3.49
35	SQ39	Suqian, Jiangsu Province	Eggplant	3.89	6.25
36	SQ34	Suqian, Jiangsu Province	Cucumber	5.06	7.83
37	SQ69	Suqian, Jiangsu Province	Pepper	3.47	5.68
38	SQ181	Suqian, Jiangsu Province	Pepper	0.77	2.04
39	SQ37	Suqian, Jiangsu Province	Tomato	1.97	6.25
40	SQ140	Suqian, Jiangsu Province	Tomato	2.94	10.37
41	SQ57	Suqian, Jiangsu Province	Pepper	7.00	10.24
42	TA134	Tai'an, Shandong Province	Pepper	1.95	5.89
43	TA13	Tai'an, Shandong Province	Pepper	2.95	3.61

44	TA61	Tai'an, Shandong Province	Tomato	4.18	5.58
45	TA121	Tai'an, Shandong Province	Cucumber	1.94	4.90
46	TA80	Tai'an, Shandong Province	Tomato	2.05	6.52
47	TA117	Tai'an, Shandong Province	Pepper	6.93	10.15
48	TA139	Tai'an, Shandong Province	Cucumber	4.73	3.71
49	TA104	Tai'an, Shandong Province	Cucumber	1.86	3.56
50	TA157	Tai'an, Shandong Province	Eggplant	2.35	4.35
51	TA54	Tai'an, Shandong Province	Pepper	1.57	3.05
52	TA28	Tai'an, Shandong Province	Tomato	2.03	3.59
53	TA115	Tai'an, Shandong Province	Cucumber	3.05	2.72

**No.**	**Strains**	**Origins**	**Hosts**	**EC_{50} against mycelial growth (μg/mL)**	**EC_{50} against spore generation (μg/mL)**
54	TA184	Tai'an, Shandong Province	Pepper	4.29	5.23
55	YT26	Yantai, Shandong Province	Tomato	6.09	7.25
56	YT02	Yantai, Shandong Province	Cucumber	2.13	5.72
57	YT39	Yantai, Shandong Province	Pepper	7.10	7.70
58	YT13	Yantai, Shandong Province	Tomato	4.86	3.34
59	YT111	Yantai, Shandong Province	Tomato	1.94	8.81
60	YT33	Yantai, Shandong Province	Pepper	2.44	6.35
61	YT68	Yantai, Shandong Province	Cucumber	1.63	3.90
62	YT74	Yantai, Shandong Province	Cucumber	2.83	4.65
63	BZ172	Binzhou, Shandong Province	Pepper	0.86	3.45
64	BZ106	Binzhou, Shandong Province	Eggplant	1.95	5.25
65	BZ82	Binzhou, Shandong Province	Pepper	2.18	2.29
66	BZ153	Binzhou, Shandong Province	Pepper	5.83	3.93
67	BZ03	Binzhou, Shandong Province	Pepper	4.72	8.08
68	BZ160	Binzhou, Shandong Province	Tomato	2.54	9.75
69	BZ75	Binzhou, Shandong Province	Pepper	1.86	4.26
70	BZ129	Binzhou, Shandong Province	Cucumber	1.58	4.81

71	BZ47	Binzhou, Shandong Province	Tomato	2.58	3.70
72	BZ157	Binzhou, Shandong Province	Pepper	2.60	3.29
73	BZ199	Binzhou, Shandong Province	Tomato	0.73	4.74
74	BZ120	Binzhou, Shandong Province	Pepper	1.77	4.78
75	BZ135	Binzhou, Shandong Province	Pepper	1.98	2.07
76	BZ103	Binzhou, Shandong Province	Pepper	5.45	3.56
77	BZ116	Binzhou, Shandong Province	Eggplant	4.40	3.87
78	XC51	Xuancheng, Anhui Povince	Tomato	2.33	8.91
79	XC04	Xuancheng, Anhui Povince	Tomato	1.68	3.48
80	XC63	Xuancheng, Anhui Povince	Pepper	2.41	4.37

**No.**	**Strains**	**Origins**	**Hosts**	**EC_{50} against mycelial growth (μg/mL)**	**EC_{50} against spore generation (μg/mL)**
81	XC164	Xuancheng, Anhui Povince	Pepper	2.37	3.19
82	XC138	Xuancheng, Anhui Povince	Pepper	2.78	4.14
83	FY132	Fuyang, Anhui Povince	Cucumber	0.83	4.08
84	FY127	Fuyang, Anhui Povince	Cucumber	1.91	4.61
85	FY112	Fuyang, Anhui Povince	Pepper	2.13	2.08
86	FY173	Fuyang, Anhui Povince	Pepper	5.74	7.38
87	FY27	Fuyang, Anhui Povince	Pepper	4.64	8.46
88	FY73	Fuyang, Anhui Povince	Pepper	2.49	3.77
89	FY32	Fuyang, Anhui Povince	Cucumber	1.82	7.04
90	FY120	Fuyang, Anhui Povince	Cucumber	1.54	4.23
91	FY197	Fuyang, Anhui Povince	Tomato	2.53	3.91
92	FY231	Fuyang, Anhui Povince	Eggplant	3.62	3.46
93	FY42	Fuyang, Anhui Povince	Tomato	1.56	5.05
94	LA154	Lu'an, Anhui Povince	Pepper	2.07	4.30
95	LA156	Lu'an, Anhui Povince	Tomato	3.04	3.50
96	LA41	Lu'an, Anhui Povince	Pepper	5.15	6.79
97	LA109	Lu'an, Anhui Povince	Eggplant	3.89	12.10

98	LA117	Lu'an, Anhui Povince	Tomato	6.94	9.24
99	LA105	Lu'an, Anhui Povince	Pepper	7.94	7.22
100	LA92	Lu'an, Anhui Povince	Pepper	0.94	5.86
101	LA13	Lu'an, Anhui Povince	Cucumber	1.76	3.29
102	LA22	Lu'an, Anhui Povince	Tomato	0.95	2.24
103	LA136	Lu'an, Anhui Povince	Pepper	1.98	3.57
104	LA09	Lu'an, Anhui Povince	Tomato	1.32	2.72
105	LA64	Lu'an, Anhui Povince	Pepper	3.06	4.97

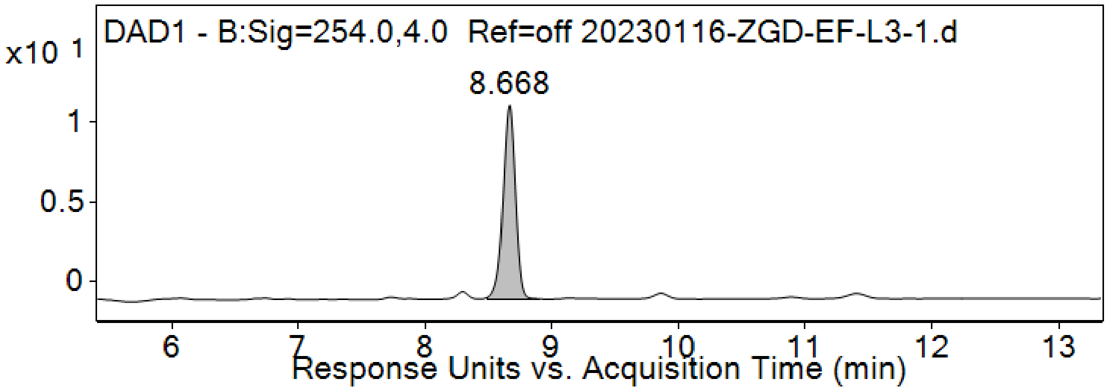

Figure S1. The UPLC chromatograms of EFL$_3$.

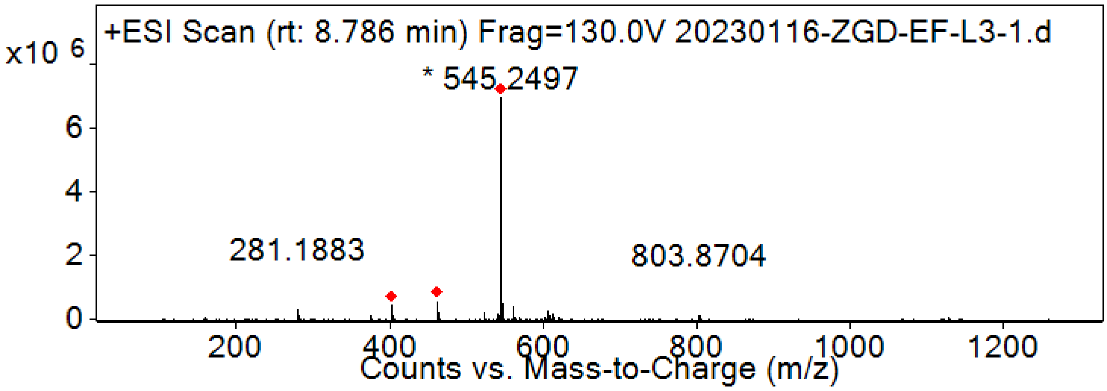

Figure S2. The HR-ESI-MS spectrum of EFL$_3$.

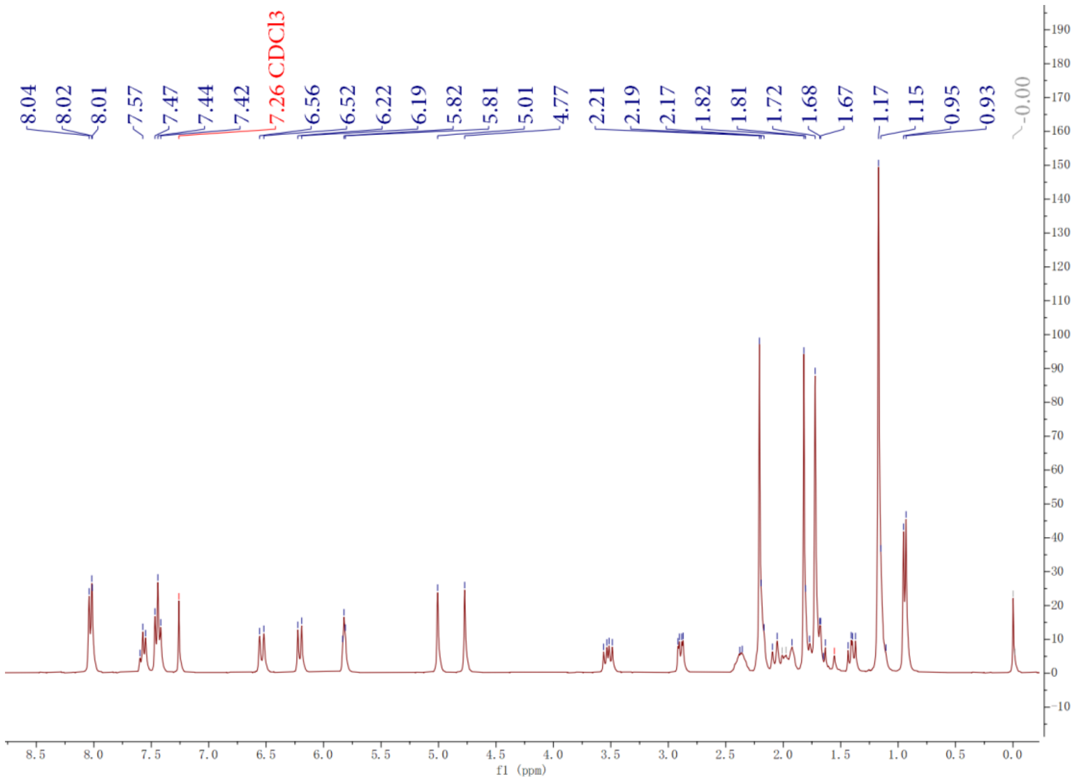

Figure S3. The ^{1}H-NMR spectrum (300 MHz, $CDCl_3$) of EFL_3.

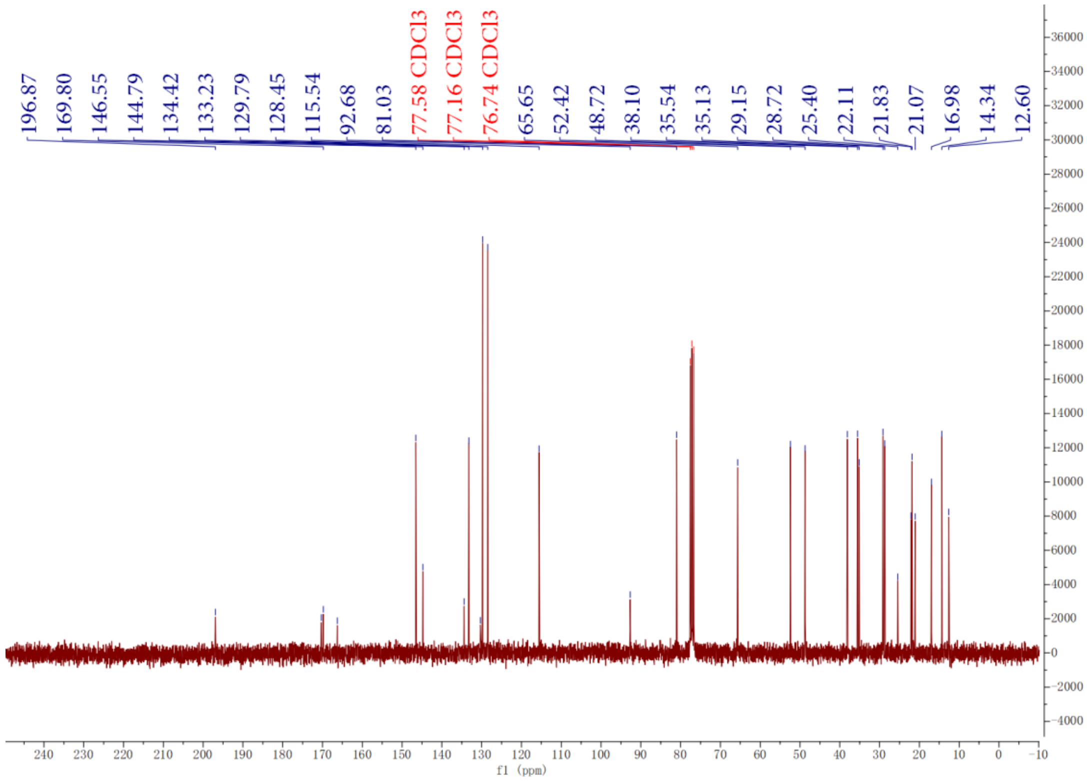

Figure S4. The ^{13}C-NMR spectrum (75 MHz, $CDCl_3$) of EFL_3.

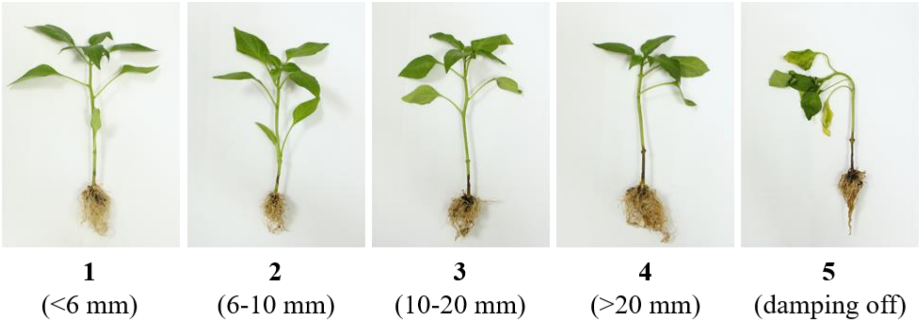

Figure S5. The disease severity of *Phytophthora capsici* on pepper plants. The disease severity was recorded at 10 d post inoculation using the crown rot system scale from 0 to 5. A scale of 0 indicates a healthy plant; 1 = symptom of less than 6 mm on the stem; 2 = 6-10 mm; 3 = 10-20 mm; 4 = symptom of more than 20 mm on the stem; 5 = damping off.
